# Building beauty: Understanding how hormone signaling regulates petal patterning and morphogenesis

**DOI:** 10.1111/tpj.70101

**Published:** 2025-03-19

**Authors:** Elena Salvi, Edwige Moyroud

**Affiliations:** ^1^ The Sainsbury Laboratory University of Cambridge 47 Bateman Street Cambridge CB2 1LR UK; ^2^ Department of Biology University of Pisa Via Luca Ghini 13 Pisa 56126 Italy; ^3^ Department of Genetics University of Cambridge Downing Street Cambridge CB2 3EH UK

**Keywords:** petals, morphogenesis, patterning, hormone Signaling, growth, polarity, differentiation, flower development, leaves

## Abstract

The corolla of flowering plants provides pivotal functions for the reproduction of angiosperms, directly impacting the fitness of individuals. Different petal shapes and patterns contribute to these functions and, thus, participate in the production of morphological diversity and the emergence of new species. During petal morphogenesis, the coordination of cell fate specification, cell division, and cell expansion is coherent and robust across the petal blade and is set according to proximo‐distal, medio‐lateral, and abaxial‐adaxial axes. However, the mechanisms specifying petal polarity and controlling cell behavior in a position‐dependent manner as petals develop remain poorly understood. In this review, we draw parallels with other evolutionarily related plant lateral organs such as leaves to argue that hormones likely play central, yet largely unexplored, roles in such coordination. By examining petal development in Arabidopsis and other angiosperms, we frame what are the knowns and the unknowns of hormones contributions to petal morphogenesis and patterning. Finally, we argue that using emerging model organisms can provide invaluable information to tackle questions that have long remained unanswered, broadening our understanding by allowing us to investigate petal morphogenesis and the tinkering of phytohormone signaling through an evolutionary lens.

## Significance Statement

Phytohormones are potent molecules that could organize a range of processes flowering plants use to shape and pattern one of their most emblematic organs, the petal. In this review, we summarize recent progress, highlight areas where data is lacking, and formulate several hypotheses that can be experimentally tested thanks to recent technological advances and the broad range of model systems now amenable to detailed functional investigations.

## INTRODUCTION

**Figure 1:**
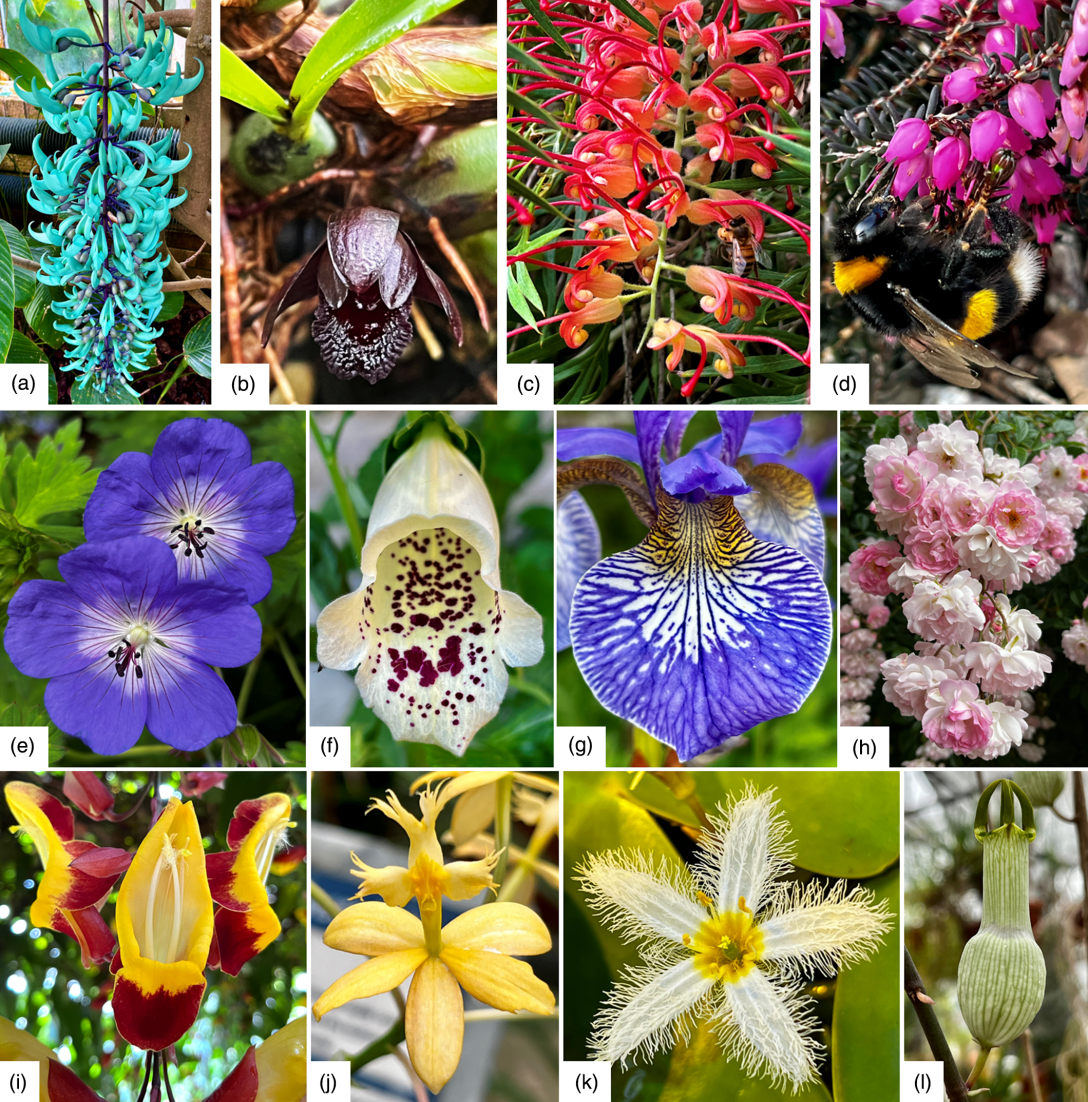
Petal morphological diversity. Selected examples of flowers from a diversity of species selected across the angiosperm phylogeny that illustrate differences in petal number, size, shape, symmetry, color (visible and UV‐range) and patterning. (a) *Strongylodon macrobotrys*, (b) *Maxillaria schunkeana*, (c) *Grevillea sp*., (d) *Erica carnea*, (e) *Geranium “Rozanne,”* (f) *Digitalis purpurea “Dalmatian Cream,”* (g) *Iris siberica*, (h) *Rosa sp*., (i) *Thunbergia mysorensis*, (j) *Epidendrum xanthinum*, (k) *Nymphoides indica*, and (l) *Ceropegia ampliata*.

Petals, collectively known as the corolla, are non‐reproductive floral organs that contribute significantly to flowers’ appearance. Petals also mediate plant–pollinator interactions and protect the reproductive organs of the flower. Hence, their morphologies are often adapted to different pollination strategies and to environments with varying conditions of UV‐B light irradiation, temperatures, and water availability (Ashworth et al., 2015; Dalrymple et al., 2020; Koski et al., 2020; Koski & Ashman, 2015; Koski & Ashman, 2016; Todesco et al., 2020). Petals number, size, shapes, and surface patterns vary widely among species, yielding a tremendous diversity of floral forms (Figure 1). Consequently, corolla characteristics are often used for taxonomical identification in *flora keys*. Petal diversity extends beyond visible traits: scent emission, humidity, and heat production are features that do not affect petal appearance but vary extensively across angiosperms and can influence pollinator behavior (Harrap et al., 2017; Harrap et al., 2021; Harrap, Hempel Ibarra, Knowles, et al., 2020; Harrap, Hempel Ibarra, Whitney, & Rands, 2020). Surprisingly, the developmental and evolutionary mechanisms that produce such morphological diversity remain largely unclear.

Petals are layered organs made of two morphologically distinct epidermises often differentially pigmented. The adaxial epidermis generally exhibits conical cells, while the abaxial cells are flatter and produce scent (Cavallini‐Speisser et al., 2021; Riglet et al., 2024). The tissue that lies in between, the mesophyll, combines parenchyma cells with specialized cell types, such as vascular bundle cells, mucilage‐producing cells, or glands synthesizing oils and volatiles (Cavallini‐Speisser et al., 2021). Although petals emerge as planar structures, they are folded during development to acquire a range of three‐dimensional shapes (Zhang et al., 2024). The diversity of petal forms at both the micro and macro scales, hence, must be produced by developmental mechanisms that control precisely in space and time both growth and differentiation of the petal cells.

Morphogenesis and patterning are two fundamental developmental processes that allow cells, tissues, and organs to acquire their shape and structural characteristics. In plants, these involve a non‐sequential combination of cell division and expansion, both as components of growth, along with cell differentiation and programmed cell death. These cellular events are spatially organized along axes marking the dorso‐ventral (adaxial‐abaxial), medio‐lateral, and antero‐posterior (proximo‐distal) polarities of the emerging organ. Hence, the nascent petal is imprinted with coordinate systems supporting the organization of different cell behaviors in space and time. To allow neighboring cells to undertake distinct developmental trajectories, boundaries are also set within or between developing organs to help generate domains with distinctive characteristics in the mature petal (Dahmann et al., 2011; Meyerowitz, 1999; Richardson & Hake, 2019; Wang et al., 2016; Žádníková & Simon, 2014). This is especially relevant in plants because position rather than lineage determines cell fate (Kim & Zambryski, 2005; Scheres, 2001).

**Figure 2:**
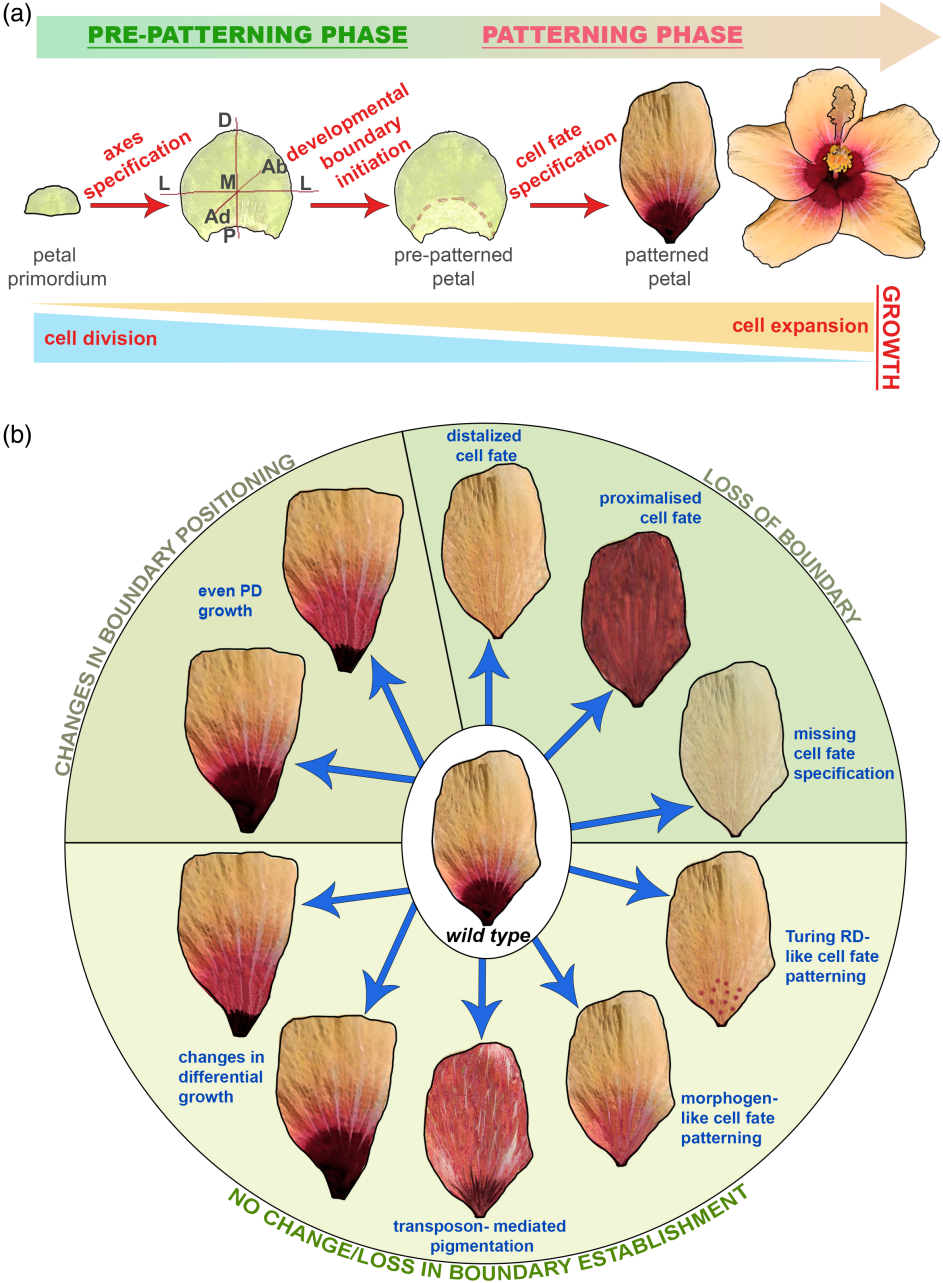
Stages of petal morphogenesis and phenotypes of a hypothetical Hibiscus petal after specific modifications in morphogenetic processes have occurred. (a) Schematic representation of key milestones of petal morphogenesis to which hormones can contribute. Ab: abaxial; Ad: adaxial; P: proximal; D: distal; M: medial; L: lateral. (b) Schematic representation of possible events leading to variation in petal patterning in relation to changes affecting developmental boundary establishment (the three different quadrants of the circle). These modifications can take place at various stages of petal development and lead to alteration of the final pattern (here the bullseye pattern of a hypothetical Hibiscus petal was used as an example).

Although petal development is a continuous process, here, we divide it into two phases (Figure 2a). Polarity axes and developmental boundaries are set during a pre‐patterning phase that precedes cell differentiation and thus takes place before the emergence of visible patterns. This pre‐patterning phase provides plant cells with positional information used during the subsequent patterning phase when differentiation takes place (Figure 2a). The patterning of developing organs relies on positional information mechanisms, such as pre‐established gradients that provide spatial cues to cells taking on differentiated fates, as well as self‐organizing processes like the Turing reaction–diffusion mechanism, which generates patterns through local activation and long‐range inhibition of patterning molecules without requiring external inputs (Gierer & Meinhardt, 1972; Green & Sharpe, 2015; Turing, 1997; Wolpert, 1969). Interdisciplinary work combining experimental data with computational modeling has played a key role in testing the biological validity of these principles (Ding, Patterson, et al., 2020; Mähönen et al., 2014; Nagashima et al., 2018; Scacchi et al., 2024; Vadde & Roeder, 2020).

Events affecting either the pre‐patterning or the patterning phase impact the final appearance of the petal: changes affecting the position of developmental boundaries during the pre‐patterning phase can modify pattern dimensions. Alternatively, differential growth can act as a “pattern modifier” by locally tuning cell proliferation and/or elongation in specific petal regions, hence changing the relative proportions of the subdomains outlined by the initial pre‐pattern (Galipot et al., 2021). Distinct patterning mechanisms can result in similar patterns in the mature flower depending on the downstream factors that interpret those patterning cues (Figure 2b). Conversely, a single mechanism can yield different patterns depending on the initial conditions of the system (for instance, the concentration of signaling molecules) or because downstream events subsequently modify the output of the patterning process (Figure 2b).

While some of the transcription factors orchestrating cell differentiation in discrete petal domains have been identified (Ballerini et al., 2019; Chopy et al., 2024; Ding, Patterson, et al., 2020; Fattorini et al., 2024; Lin & Rausher, 2021), little is known regarding the upstream pre‐patterning processes that set developmental boundaries in petal primordia and restrict the activity of those transcriptional regulators to subdomains of the corolla. Plant hormones evolved in unicellular organisms as signals to integrate responses to external stimuli with growth processes, becoming developmental regulators *per se* during the acquisition of multicellularity and the diversification of land plants (Fiedler & Friml, 2023; Powell & Heyl, 2023). Originally, this solution could have been selected because of the chemical properties of the hormone molecules. An integral characteristic of hormones is their ability to be transported and differentially distributed in a time‐tuned manner, allowing the establishment of local concentration gradients. Co‐option, the evolution of additional components, elaboration of sophisticated transport and homeostasis systems and configuration of new gene regulatory networks have further expanded an already adaptable, tuneable, and redundant hormonal signaling system. This contributed to establishing hormones as recurrent instructors of morphogenetic programs and growth regulators (Alabadí et al., 2009).

Hence, it is tempting to hypothesize that hormones are paramount agents allowing petals to develop a multitude of patterns and shapes. Here, we review the evidence that supports this hypothesis from different angles. In Section 2, we investigate the role hormones could play in tracing the boundaries that separate neighboring domains in developing petals to eventually produce a patterned organ. In Section 3, we examine the contributions of hormonal signaling to cell differentiation during the later phase of corolla development. Petals enlarge significantly to reach their mature shape and size: in Section 4, we consider the idea that, as long‐distance messengers and growth regulators, hormones could mediate communication between tissues and act as signals that coordinate growth between the different petal regions to either conserve or modify patterns that have been laid out during early pre‐patterning stages. In Section 5, we explore whether modifications of hormone signaling during evolution facilitated petal adaptation to ever‐changing environments, generating diversity. This helps us delve into the causes of the many petal forms to illuminate the possible paths leading to corolla evolution in Section 6.

## HORMONE CONTRIBUTION TO AXES AND DEVELOPMENTAL BOUNDARIES ESTABLISHMENT IN PETALS

Plant hormones, especially auxin, are essential to initiate petal primordia and specify where those emerge on the floral meristem (FM) (Lampugnani et al., 2013) (Figure 2a). These hormonal response patterns are likely inherited from the FM by the primordium, where they are then propagated and dynamically regulated to set up the axes of the nascent petals (Bossinger & Smyth, 1996; Brewer et al., 2004; Griffith et al., 1999; Huang & Irish, 2024; Lampugnani et al., 2013; Takeda et al., 2022). As hormonal responses are central to coordinating the activities of the shoot apical meristem (SAM) and the FM (reviewed in (Shi & Vernoux, 2022)), invoking a role for hormones in specifying axes and initiating developmental boundaries to prepattern growing petal primordia is a straightforward hypothesis, even if no report has demonstrated this yet. Hints that phytohormones could fulfill such roles come from studies that connect hormones to boundary establishment in other plant organs (Wang et al., 2016) and data showing that hormones initially steer polarity in the growing petal (Sauret‐Güeto et al., 2013).

### Auxin, a positional signal that steers the polarity of growing petals

Evidence suggests that differential distribution spatially guides the growth of petal primordia. At petal initiation sites across the FM, the expression of the auxin efflux carriers of the PIN‐FORMED family (PIN) is partly regulated cell‐autonomously by RABBIT EARS (RBE), a C2H2 zinc finger, and non‐cell‐autonomously by PETAL LOSS (PTL), a trihelix transcription factor. Mutations in those genes prevent the normal initiation, orientation, and subsequent development of petals by disrupting auxin response. In *ptl* mutants, petal identity is not lost, but the boundary between sepals is disrupted, affecting the region where petals are specified (Brewer et al., 2004; Lampugnani et al., 2012). The mechanism involving *PTL* function in parallel with those specifying petal organ identity (Griffith et al., 1999) regulates primordia growth and proximal polarity after petal specification. Later in Arabidopsis petal development, the auxin response reporter *DR5:GUS* is active only in distal spots on the petal edge and in pro‐vasculature sites (Aloni et al., 2006). Analysis of *DR5:GFP* at different petal developmental stages coupled with computational modeling expanded further those observations, showing that at the earliest stage of development, the auxin response marks the petal tip (Sauret‐Güeto et al., 2013). Later, the auxin response propagates divergently from the main longitudinal axis along the petal edges of the primordium, following the instructions of a modeled distal organizer and acting as a marker for the petal distal polarity and the growth field until late development, when the *DR5:GFP* signal fades. The fact that *pin1* and *pin6* mutants in Arabidopsis have altered petal morphology supports the role of auxin signaling in establishing polarities for petal growth (Bender et al., 2013; Okada et al., 1991). Notably, in Arabidopsis, the transcriptional repressor JAGGED (JAG) is a target of the flower organ identity MADS‐box genes AGAMOUS, SEPALLATA 3, and APETALA 3 (Gómez‐Mena et al., 2005; Kaufmann et al., 2009; Ó'Maoiléidigh et al., 2013; Wuest et al., 2012) and is expressed in the distal petal region where it binds the promoter of genes regulating growth, like auxin response regulators and the cell‐cycle inhibitor KRP2 (Schiessl et al., 2012). JAG seems to act as the distal organizer for petal growth, linking organ identity specification with polarity control of cell proliferation and expansion rates. By repressing *PTL*, which emerges as the polarity proximal organizer in computational simulations, JAG indirectly controls auxin efflux and transport, establishing overall organ polarity and driving petal growth (Sauret‐Güeto et al., 2013). Whether auxin functions as the effector of petal polarity can be expanded across the angiosperms and whether it fulfills this role alone or in concert with other phytohormones remain to be explored.

### Hormones are mobile signals that could establish petal developmental boundaries

Genetic regulators of various petal features have been identified (see Section 3). Because their action is restricted to subregions of the petal primordium, these likely act after developmental boundaries are specified, implying that petals must be pre‐patterned before those factors come into play. Data from three recent studies support the hypothesis that pre‐patterning mechanisms involving unidentified mobile signals establish developmental boundaries early during petal development (Chopy et al., 2024; Ding, Patterson, et al., 2020; Riglet et al., 2024). These mobile signals can be hypothetically hormones, small peptides, mRNAs, lipids, other molecules, or a combination thereof.

In *Petunia hybrida*, the limb and tube that make the corolla can be specified modularly and independently depending on the spatio‐temporal expression of the B‐class MADS‐box gene *PhDEFICIENS* (PhDEF) in the primordium (Chopy et al., 2024). This indicates that the underlying programs that PhDEF sets in motion are restricted by developmental boundaries established earlier by unidentified mobile molecules (see Section 4). In *Mimulus lewisii*, a reaction–diffusion mechanism creates an anthocyanin‐spotted pattern on the petal nectary guides. In this system, RED TONGUE (RTO) acts as an inhibitor of anthocyanin synthesis, and the *rto* mutant develops a fully pigmented nectar guide, but ectopic anthocyanin production is restricted to the proximal petal domain, not expanding to the adjoining distal region (Ding, Patterson, et al., 2020). This suggests that the Turing‐like mechanism is spatially restricted to the petal base and overlaid on a petal pre‐patterned by a putative positional information system, which delineates a boundary separating the nectary‐bearing proximal region from the petal lobe (Ding, Patterson, et al., 2020). In Venice mallow (*Hibiscus trionum*), which produces flowers with a striking bullseye pattern, petal pre‐patterning and the emergence of boundary cells have been directly visualized (Riglet et al., 2024). In this species, the behavior of adaxial epidermal cells is already programmed along the petal primordium proximo‐distal axis long before its cells acquire their characteristic colors, shapes, and textures. Distal cells actively divide while proximal cells experience anisotropic elongation, with the largest cells found where the future bullseye boundary, macroscopically visible only later in development, will emerge (Riglet et al., 2024). Epidermal cells on either side of the boundary subsequently acquire distinct shapes and textures and produce different mixtures of flavonoid pigments, resulting in purple, anthocyanin‐rich tabular striated cells in the proximal petal and off‐white, flavonol‐rich conical smooth cells in the distal petal (Moyroud et al., 2022). Overall, it remains unclear whether the yet‐to‐be‐identified mobile signals regulating the establishment of those boundaries share the same nature and function as hormones. However, given the extensive roles that hormones play in plant development, they constitute strong candidates, as evidenced by their involvement in patterning other lateral organs (see Section 6).

## CONTRIBUTIONS OF HORMONE SIGNALING TO PETAL CELL FATE SPECIFICATION AND DIFFERENTIATION

The colorful motifs present on the corolla of most flowering plants are generated by restricting pigment synthesis to specific petal cells, by adjusting their composition and concentrations, or by combining different pigment molecules in distinct petal regions (reviewed in Fairnie et al., 2022). Beyond pigmentation, the diversity of epidermal cell shape and texture found along the proximo‐distal axis epitomizes the relationship between petal morphology and its eco‐physiological functions. In *Petunia hybrida*, conical cells are present in distal limbs, predominantly on the adaxial surface where they capture light and intensify pigments, assist in pollinator grip, and reduce wettability (reviewed in Moyroud & Glover, 2017). At the most distal part of the petal tube, instead, elongated cells bear waxy cuticular striations (Cavallini‐Speisser et al., 2021). Work in other species has shown that striations on top of flat tabular cells can diffract light to produce an iridescent halo in the UV‐blue part of the light spectrum (Moyroud et al., 2017), visible to pollinating insects. Close to the base of the tube, epidermal cells exhibit a pronounced central papilla and could enhance scent release or exert other functions as specialized metabolite secretion during pollinator visits (Cavallini‐Speisser et al., 2021). Hence, patterning mechanisms must be in place to specify cell fate and differentiation in a robust and precise fashion across the petal epidermis. Evidence suggests such processes are at least in part under hormonal control.

### Hormones can control pigment production in petals

Gibberellins (GAs) regulate petal pigmentation by promoting anthocyanin synthesis in *Petunia hybrida* flowers (Weiss, 2000; Weiss et al., 1992; Weiss et al., 1995; Weiss & Halevy, 1989). Adding hormones to *in vitro* cultures of flower buds also influences pigment production (Liu et al., 2020): gibberellic acid enhances carotenoid and chromoplast production in plant cells, while abscisic acid (ABA) and ethylene suppress it (Vainstein et al., 1994; Vishnevetsky et al., 1997). Metabolomic studies recently uncovered a correlation between pigmentation changes and endogenous levels of auxin, CKs, GAs, brassinosteroids (BRs), jasmonate (JA), ethylene, and ABA, implying that hormones may regulate both physiological and developmental changes in flower color, but the associated regulatory mechanisms are still obscure (Huang et al., 2024; Xia et al., 2021). In ripening fruits, several hormones, like ABA, ethylene, JA, BR, auxin, and CKs, were shown to regulate the expression of MYB, bHLH, and WD40 (MBW) factors involved in the transcriptional regulation of the flavonoid pathway, a class of multipurpose molecules that includes red‐to‐blue anthocyanins and cream‐yellow flavonols (reviewed in Wang et al., 2023), but whether this is also the case in developing petals remains to be tested. Transcription factors of the R2R3‐MYB family, like DEEP PURPLE and PURPLE HAZE, regulate anthocyanin production in Petunia flowers, and their expression is under both environmental and developmental control; however, their regulation by hormonal pathways has not been elucidated to date (Albert et al., 2011). As hormones can regulate changes in pigmentation induced by environmental cues like light (reviewed in Li & Ahammed, 2023; Shi et al., 2023), phenomena such as the “bud‐blush,” where regions of petals exposed to light develop anthocyanin pigmentation, are likely vestiges of an ancestral mechanism controlling pigment production upon stress induction. This hints that phytohormones could have gained a developmental role during evolution to possibly contribute to the production of robust petal pigmentation patterns.

### Hormones participate in the specification and elaboration of petal cell structural features

Like many angiosperms, Arabidopsis flowers produce striated conical cells in their distal region. Past studies have shown the importance of transcription factors like MIXTA (Glover et al., 1998) and SHINE (Li‐Beisson et al., 2009; Shi et al., 2011), structural proteins like katanin p60 (Ren et al., 2017), and transporters of cuticular lipids like ABCG13 (Panikashvili et al., 2011) for the formation of the conical cell shape and striated texture, respectively. We propose that hormones could contribute to the regulation of cell morphology in two ways: by controlling the expression of genes that govern cell growth and cuticle production and by directly impacting the mechanical processes that shape cells and their texture.

According to the *acidic growth theory*, auxin mediates the apoplastic acidification necessary for cell expansion (Arsuffi & Braybrook, 2018). Conical cells are initially flat and later bulge out to become cone‐shaped. In Arabidopsis, apoplastic pH dynamics during such cell expansion correlate with changes in auxin response: conical cell outgrowth and tip sharpening are associated with decreased apoplastic pH and increased auxin signaling (Dang et al., 2020). Whether auxin directly contributes to cell shape elaboration by changing cell wall properties through acidification or whether auxin signaling activates the expression of regulators and structural genes involved in the specification of conical cell fate in the distal region remains to be understood. In the first case, it is interesting to investigate how auxin interprets spatial information from the proximo–distal axis and developmental boundary and translates it into pH readouts. In the second case, it is important to identify the molecular players that relay the auxin signal to regulate cell differentiation. Notably, double mutants of the auxin response factors *arf6, arf8* show conical cell expansion defects in Arabidopsis; hence, ARF6 and ARF8 could be the missing link between the auxin‐mediated specification of growth directions and cell morphology differentiation in Arabidopsis (Dang et al., 2020; Sauret‐Güeto et al., 2013). The spatial control of petal cuticle patterning depends on the direction and extent of cell growth but also on the amount of cuticle produced and its chemical composition (Moyroud et al., 2022). Several hormones contribute to petal cell expansion (see Section 4) and are thus likely to impact, at least indirectly, the texture of the cell. Phytohormones could also control cell texture directly by regulating the expression of genes involved in cuticle assembly. However, whether this is the case remains to be investigated.

## HORMONES CONTROL COROLLA GROWTH AND PETAL PATTERN DIMENSIONS

The regulation of growth is central to petal development and its ecological relevance: growth controls the size and shape of petals as well as the proportions of the colorful motifs on the corolla epidermis, all features that directly impact plant fitness (reviewed in Fairnie et al., 2022; Woźniak & Sicard, 2018). Growth, encompassing both cell proliferation and cell expansion, occurs continuously throughout petal development, from primordium specification to anthesis. Growth is also fundamental because the elements that pre‐pattern the petal primordia (polarity axes and developmental boundaries; see Section 2) are set very early during development, long before the corolla reaches its mature size (Riglet et al., 2024). Therefore, growth can act as a “pattern modifier” by changing the relative sizes of the petal regions set during the pre‐patterning phase (Galipot et al., 2021; Riglet et al., 2024). Hormones are growth regulators by definition: they act on cell proliferation and cell expansion, also orchestrating the transition between the two and coordinating growth with differentiation across the different petal regions. Hence, to understand the contribution of hormones to petal morphogenesis and patterning, it is important to examine carefully in space and time their ability to fine‐tune all aspects of growth.

### Hormones control cell proliferation, cell expansion, and the transition from one to the other during petal development

GAs were first detected in flower corollas in the late 1960s (Harris et al., 1969), where they promote petal elongation: in Arabidopsis, the GA biosynthesis mutant *ga1‐1* has arrested petal growth, with thin and “scaly” petals, which can be rescued by the application of GAs (Cheng et al., 2004; Goto & Pharis, 1999). In cucumber, the development of female flowers depends on the translocation of GA precursors from the ovary to the sepals and petals, where it is converted to the bioactive form before the rapid petal growth phase that precedes anthesis. Unexpectedly, *GID* and *DELLA* genes, respectively coding for GA receptors and repressors, are highly expressed in all floral parts at all developmental stages, suggesting that the GA‐mediated control of petal growth occurs mainly through the translocation and regulation of GA levels or through post‐transcriptional regulation of GID and DELLA proteins (Pimenta Lange & Lange, 2016).

Other phytohormones participate in the control of petal growth. A genome‐wide association study between two accessions of *Brassica napus* differing in petal size uncovered two regulators of cell division: a homologue of *RAP2.2*, a potential inhibitor of the cell cycle in response to ethylene, and *ARABIDOPSIS RESPONSE REGULATOR 4* (*ARR4*), a CK‐induced response regulator negatively transducing the CK signal and potentially accelerating the cell cycle to increase petal size (Qian et al., 2021). Functional investigations support the idea that CK homeostasis is also central to petal growth. In Arabidopsis, the double mutant for the catabolic CYTOKININ OXIDASES 3 and 5 produces larger petals than wild‐type flowers, indicating that elevated CK levels delay cellular differentiation and/or extend the division window or promote faster cell division rates (Bartrina et al., 2011). In rose (*R. hybrida*) petals, knocking down miR159 causes transcript accumulation of its target *CKX6*. This promotes CK clearance leading to a shortened cell division period and smaller petals. Conversely, increasing the amount of CK by mutating C*KX6* induces a prolonged cell division window, phenocopying the effects of CK application. *RhMIR159* expression is modulated by histone H3 lysine 9 acetylation of its promoter, which is controlled by a complex made of the R2R3‐type MYB RhMYB73, the co‐repressor RhTOPLESS, and the histone deacetylase RhHDA19 (Jing et al., 2023). This ensures proper timing to exit the cell division phase through an interdependent post‐transcriptional and epigenetic regulation of CK catabolism.

CKs also act as switches from cell proliferation to cell expansion during petal development. CKs content declines during petal development in roses, and this coincides with the arrest of cell proliferation (Wang et al., 2024). Silencing the CK‐responsive transcription factor RhRAP2.4 L reduces petal size due to decreased cell number and cell size, suggesting a premature transition from proliferation to expansion. RhRAP2.4 L directly binds the promoter of the cell cycle regulator *RhKRP2* and the cell expansion regulator *RhBIG PETALub*, a gene encoding a bHLH transcription factor, respectively up‐ and down‐regulating them. This dual action guarantees a coordinated progression through the growth phase by synchronizing the transition from cell proliferation to cell expansion (Wang et al., 2024).

JA controls the expression of *BIGPETAL* (*BPE*), which yields bigger petals with an abnormal venation pattern when non‐functional. In the *opr3* JA synthesis mutant, petal size increases due to enhanced cell expansion, akin to *bpe‐1*, because *BPE* expression is downregulated (Brioudes et al., 2009). In fact, JA regulates cell expansion by favoring the alternative splicing variant of BPE, BPEp, involved in petal development. The auxin response factor ARF8 can interact with BPEp, and together they affect the expression of auxin‐responsive genes. This leads to decreased auxin sensitivity in petal cells, which in turn affects cell expansion and growth patterns (Brioudes et al., 2009; Varaud et al., 2011). Single and double mutants indicate that both ARF8 and BPEp restrict petal growth throughout development by regulating cell expansion. Additionally, ARF8 influences cell proliferation at the initial stages of flower development, suggesting ARF8 functions are time‐ and interactor‐dependent (Brioudes et al., 2009; Varaud et al., 2011).

Recent results substantiate the idea that ARF8‐mediated auxin signaling is a major contributor to petal growth and morphogenesis. Nectar‐containing spurs are petal tubular extensions that evolved independently multiple times across the angiosperms. *AqARF6* and *AqARF8* are highly expressed in Columbine spurs, and single and double mutants for these genes produce shorter floral organs, with a reduced petal spur length due to decreased anisotropic cell expansion (Zhang et al., 2020). The shift from cell division to anisotropic cell expansion is the primary force driving petal spur elongation in *Impatiens uliginosa* (Li et al., 2024) and in Aquilegia (Puzey et al., 2011) with input from brassinosteroids (BRs). Disturbance of BR signaling leads to abnormal spur morphologies, and the application of the BR analogue brassinolid (BL) affects anisotropic cell elongation in the lower half of the spur. The differential response to BR along the spur correlates with a natural gradient in cell elongation, signifying a spatial regulation of BR distribution, sensitivity, and/or signal transduction components within the petal tissues (Conway et al., 2021). Hence, the phytohormone's ability to sculpt petal shape could be guided by the pre‐pattern outlined during the early phase of corolla development.

Because multiple hormones are involved in controlling petal growth, extensive signaling integration must take place. In *Gerbera hybrida* (Asteraceae), the TCP class I transcription factor GhTCP7 interacts with GhWIP2, a zinc finger protein, to repress petal expansion. This interaction widens the functionality of individual proteins by changing their ability to bind the promoter of *GhIAA26*, a repressor of the auxin transcriptional response that suppresses cell and ray floret expansion (Ren et al., 2023). Interestingly, GA and ABA antagonistically regulate *GhWIP2*, while auxin represses both *GhTCP7* and *GhWIP2* (Ren et al., 2018). Hence, the GhTCP7–GhWIP2 protein complex represents a nexus for the crosstalk between GA, ABA, and auxin, contributing to petal growth. Whether this complex is specific to some members of the daisy family or whether it is conserved across the angiosperms remains to be established, but it constitutes an elegant molecular mechanism allowing phytohormones to act in concert to shape the corolla.

### Hormones can coordinate growth and cell differentiation during corolla patterning

How growth and differentiation across the petal are connected to each other during the patterning phase is poorly explored, but observations in some species suggest that phytohormones act as coordinators. In petunia, GAs stimulate both corolla growth and anthocyanin synthesis, but they appear to do so using distinct mechanisms (Weiss & Halevy, 1989). Anthers, a site for GA synthesis, are necessary for the initiation of both growth and pigmentation in the initial stages of corolla development. Stamen removal causes stunted and depigmented adjacent petals, while externally applied GAs compensate for the absence of anthers. However, kinetic studies with the GA biosynthesis inhibitor paclobutrazol (PAC) demonstrated independent regulation of growth and anthocyanin synthesis, suggesting they are unrelated mechanisms or that distinct GA concentration thresholds trigger these two processes. GAs are primarily active during the induction phases of pigmentation and growth, while in the subsequent rapid growth stages, exogenous GA and stamen presence become less important. However, such a dual role for GAs may not be applicable across the angiosperms: in snapdragon, anther‐derived GAs promote petal expansion but are not required for pigmentation, pointing to a divergence in the gene regulatory networks (GRN) that coordinate growth and differentiation between different tissues of the flower (Shang et al., 2011).

### Hormones possibly coordinate growth between the different petal tissue layers

As long‐distance messengers, hormones can coordinate the morphogenesis of distinct cell layers within an organ. In Petunia corolla, tube and limb cells are specified by the spatial‐ and time‐resolved activity of PhDEF, a non‐mobile transcription factor that is predicted to trigger unknown cell non‐autonomous signals acting downstream to coordinate the differentiation of the neighboring tissue layers (Chopy et al., 2024). Hormones certainly represent promising candidates for such signals because of their ability to move between cell layers and because they fulfill similar functions in other organs, like for BRs in leaves and roots (Graeff et al., 2020; Hacham et al., 2011; Savaldi‐Goldstein et al., 2007; Zhiponova et al., 2013). Furthermore, hormones and their downstream effectors can act alongside mechanical interactions between cells to ease the coordination between parts of a growing organ (Heisler et al., 2010), as demonstrated for BR in Arabidopsis and Utricularia stems (Kelly‐Bellow et al., 2023). Nonetheless, unequivocal demonstrations of this scenario remain challenging, particularly if the same key players control coordination and growth, and alterations of one mask the effects on the other.

### Two faces of the same coin: Hormones could maintain or modify pre‐pattern proportions by controlling growth locally

The early establishment of pre‐patterns in petal primordia raises the question of how pattern proportions—and thus the relative sizes of the different petal regions—are maintained while petals often grow exponentially. This is crucial as petal proportions can directly impact pollinator attraction or plants’ ability to cope with abiotic factors (Koski & Ashman, 2015; Riglet et al., 2024; Todesco et al., 2020). In Venice mallow, the proximal and distal petal domains grow at the same overall rate, maintaining bullseye proportions while the petal experiences a 100‐fold size increase. While growth is powered by cell divisions in the distal region, the proximal domain grows mostly through cell expansion (Riglet et al., 2024). In the Arabidopsis root meristem, auxin acts as a size coordinator between two distant zones, the stem cell niche and the cells transitioning to cell elongation/differentiation (Moubayidin et al., 2013). A similar hormone‐mediated mechanism could ensure coordinated growth between the two bullseye regions via inter‐domain hormone transport, local signaling, and homeostasis, but this has not yet been tested. Whether evolutionary tinkering with phytohormone signaling can modify petal pattern proportions by altering the coordination of the different petal regions also represents an interesting direction for future investigations.

Alternatively, plant hormones could modify petal pattern proportions by controlling growth locally. Indeed, boundary establishment creates developmental modules capable of growing and differentiating independently from each other (see Section 2). Theoretically, this allows growth regulators to act locally, changing the size and geometry of a given petal domain regardless of which developmental trajectories are undertaken by the rest of the corolla (Galipot et al., 2021). Columbine petal spurs develop in two phases: an initial phase of localized cell divisions to generate the spur, followed by a phase dominated by cell elongation that determines the final spur length (Zhang et al., 2020). To shape the spur, a specific sub‐domain within the petal blade must be individualized, and its growth must be controlled independently from other petal domains. This is, at least partly, directed by the spatial restriction of BR signaling. RNA‐seq data of the early stages of spur primordia found two BR‐related genes preferentially expressed in the nascent spur: a homologue of *AtDWARF4* (*DWF4*), a cytochrome P450 active in the BR biosynthesis pathway, and a homologue of the *AtBRI1‐EMS‐SUPRESSOR1* (*BES1*) and *AtBRASSINAZOLE‐RESISTANT1* (*BRZ1*) paralogues. Tuning down their expression via Virus‐Induced Gene Silencing (VIGS) causes defects in spur morphology (Conway et al., 2021). Likewise, BR acts on cell elongation in *Gerbera hybrida* ray floret petals (Huang et al., 2017; Lin et al., 2021). Nothing is known about the specific domains of action of BR in this species, but BR treatments affect gene expression and morphology mostly in the basal region of the ray floret, unveiling a regionalization in petals ability to sense and/or respond to hormone signaling (Ren et al., 2018). Such regionalization was also observed in Venice mallow, where *TCP4* homologs are preferentially expressed in the distal region of the petal, and constitutive overexpression of *HtTCP4.1* triggers excessive cell division uniquely in the proximal region, yielding flowers with a larger bullseye. Whether the effect of HtTCP4.1 on cell division is mediated by hormones remains to be investigated. Altogether, these results demonstrate that perturbing growth locally, via changing the expression of a single gene or hormone signaling, can modify pattern proportions after pre‐pattern establishment (Ren et al., 2018; Riglet et al., 2024).

## HORMONAL SIGNALING PATHWAYS AS EVOLUTIONARY TARGETS TO DIVERSIFY PETAL MORPHOLOGY

Hormone signaling likely plays a leading role in the evolution of floral traits, facilitating the emergence of natural variation (change in trait state) and even the emergence of novelty (creation of new traits) (Wessinger & Hileman, 2020). Here, we review the evidence to date supporting the idea that evolution innovates by manipulating hormonal signaling pathways.

Tubular corollas are commonly found across the angiosperms: more than 80,000 species produce tubular flowers, and this trait has independently evolved multiple times during Angiosperm history (Ding, Xia, et al., 2020). Tube‐shaped flowers restrict access to nectar and pollen rewards, enabling the formation of new or more specialized plant‐pollinator relationships—hence, it is likely to have facilitated the diversification of Angiosperms and to have promoted speciation (Fenster et al., 2004). In Lewis’ monkeyflower (*Mimulus lewisisii*) corolla tube formation results from the synchronized growth between the petal primordium base and the petal inter‐primordial region, and auxin response through *MlARF4*, the ortholog of *AUXIN RESPONSE FACTOR 4*, is necessary for this synchronized growth. In the wild type, the DR5 auxin response reporter peaks at the initiation sites of individual petal primordia. The DR5 signal is later detected in the inter‐primordial regions that grow upward synchronously around the entire circumference of the petal whorl, generating the corolla tube. *flayed* mutants present unfused petals due to mutations in orthologs of ARGONAUTE 7 and SUPPRESSOR OF GENE SILENCING 3 (AtAGO7 and AtSGS3) which contribute to the maturation of TAS3‐derived tasiRNAs. These post‐translationally repress the expression of *MlARF4* and its close relative *MlARF3* during the initial stages of corolla tube formation. The increased stability of MlARF3/4 mRNAs in *flayed2* disrupts the auxin response, inhibiting lateral expansion at the petal base and arresting upward growth of the inter‐primordial regions, eventually yielding unfused petals (Ding, Xia, et al., 2020). This pathway suggests that auxin signaling is central to the production of both fused and unfused corollas, given the extensive occurrence of the TAS3‐ARF4 module across the plant kingdom (Xia et al., 2017) and it will be interesting to test if gains and losses of petal fusion events occurred through replicated evolution targeting auxin signaling across the angiosperms.

Morphological divergences between closely related species can be exploited to gain insights into evolutionary trajectories. Indeed, comparing *A. thaliana* and *Cardamine hirsuta* led to key findings regarding the evolution of simple and compound leaves (Vlad et al., 2014). Petals of *Hibiscus richardsonii* and its sister species *H*. *trionum* are similar in size and overall shape, but the corolla of *H. richardsonii* harbors a reduced bullseye. This is due, in part, to the bullseye boundary being specified closer to the petal base during the pre‐patterning phase (Riglet et al., 2024). By leveraging the possibility of a direct comparison of the molecular mechanisms regulating their pre‐patterning and growth, it will be possible to observe the role(s) phytohormones may play in maintaining petal shape and size while allowing variation of the bullseye boundary positioning, either through modification of pre‐patterning events or later through local changes in growth with hormones acting as pattern modifiers (Figure 2b). Particularly interesting is the opportunity to link changes in GRNs and their adaptive significance in response to the selective pressures organisms are subjected to. Indeed, several studies have shown how spur presence/absence and changes in its length affect pollinator identity and participate in diversification and speciation (Fernández‐Mazuecos et al., 2019; Whittall & Hodges, 2007). A comparative transcriptomic analysis between the spurless *Antirrhinum majus* and the spurred *Linaria vulgaris* revealed that genes related to CK and auxin biosynthesis as well as GA response were more expressed in Linaria petals, suggesting a connection between those hormones and spur outgrowth (Cullen et al., 2023). In Columbine, spur formation is associated with the activity of POPOVICH, a C2H2 zinc finger transcription factor that promotes localized cell division. Genetic variation at the *POP* locus accounts for the presence or absence of spurs in *A. coerulea* and *A. ecalcarata*, respectively (Ballerini et al., 2019; Ballerini et al., 2020). Whether *POP* expression is under hormonal control and/or whether its activity is facilitated by hormonal signaling is not known, but interestingly, POP is closely related to RBE, a known modulator of auxin activity during petal initiation (Lampugnani et al., 2013). These cases illustrate how evolutionary tinkering with hormone pathways, especially auxin signaling, could mediate petal shape diversification, directly affecting abiotic and plant‐pollinator interactions.

## COMMON ANCESTRY COULD ACCOUNT FOR SHARED PHYTOHORMONE PROCESSES THAT SHAPE AND PATTERN ALL LATERAL ORGANS OF FLOWERING PLANTS

According to the foliar theory formulated by Wolff and Goethe in the XVIII century and revived more recently (Coen, 2001; Kaplan, 2001), flower organs are “transformed leaves.” As such, the mechanisms and GRNs that serve in leaf development are likely to have been repurposed in floral organs like petals. Leaf developmental studies could thus inform research efforts and fast‐track discoveries related to petal development.

**Figure 3:**
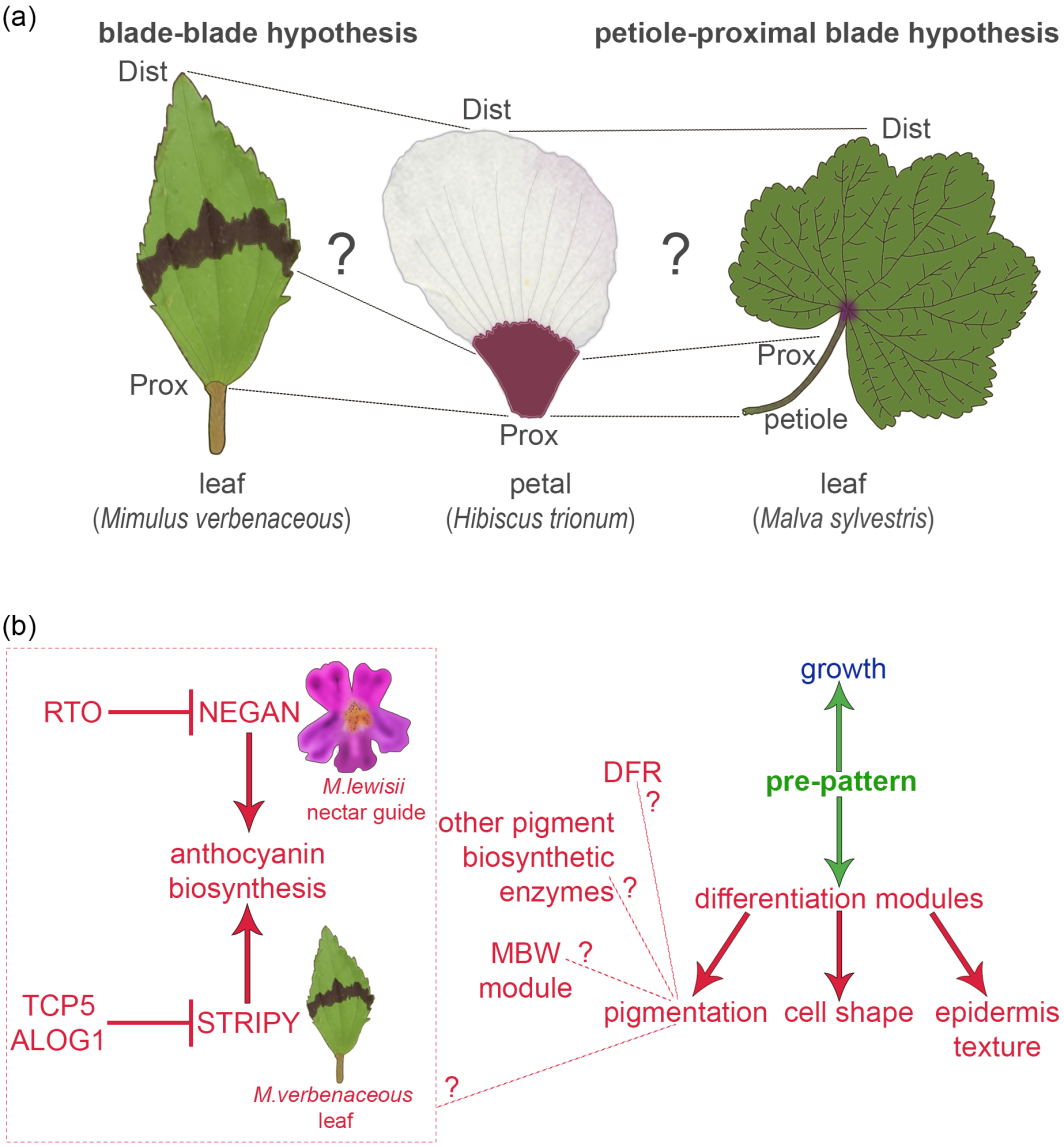
Comparative approaches highlight the role of novel model systems to formulate testable hypotheses and further our understanding of the mechanisms governing petal evolution and development. (a) Parallels in the structure of the leaves from *Mimulus verbenaceous* and *Malva sylvestris* and the petal of *Hibiscus trionum* point out putative equivalent regions (i.e., on the left: leaf proximal region —> petal proximal region; on the right: petiole —> petal proximal region). Prox: proximal; Dist: distal. (b) Mechanisms governing pre‐patterning (green) and growth (blue) could be largely shared between leaves and petals, while the downstream genetic modules controlling cell fate specification and differentiation (in red) could be specific to each organ type. Feedback and crosstalk within and between the key steps represented are likely but have been omitted for clarity. The left rectangle provides an example where localized pigment production to generate colorful patterns on the petal of *Mimulus lewisii* and the leaf of *Mimulus verbenaceous* relies on distinct sets of transcription factors (Ding, Patterson, et al., 2020; LaFountain et al., 2024; Yuan et al., 2014).

However, how much is shared and how much is unique to leaf developmental programs and those governing floral organ formation remains to be established. This is due, in part, to the difficulty of resolving deep homology. Comparing equivalent structures is essential to identify the shared roles hormones play in controlling the development and evolution of leaves and petals, but it is still unclear how the different parts of both organs relate to each other: as both planar organs, we propose their proximal and distal regions could be equivalent to each other (blade‐blade hypothesis, Figure 3a). Alternatively, the leaf petiole could be homologous to the petal proximal region, while the leaf blade would correspond to the distal petal domain (petiole‐proximal domain hypothesis, Figure 3a). Progress has also been hindered due to a lack of systematic examinations: often, studies reporting defects in leaf development do not discuss possible phenotypes in floral organs and vice versa. In leaves, the determination of adaxial‐abaxial polarity takes place before primordia emerge from the SAM (reviewed in Choudury & Husbands, 2023). It involves auxin response, alongside transcription factors, miRNAs, and tasiARFs; however, whether adaxial‐abaxial petal polarity is also affected when these factors are impaired still needs to be tested.

Below we discuss a few representative cases hinting that the processes that shape and pattern plant lateral organs during both pre‐patterning and patterning phases represent complex combinations of old modules inherited from ancestral mechanisms and new elements specific to each organ type.

### Hormonal regulation of polarity axes and boundary establishment: Parallels between leaves and petals

Class II *TCP* genes are critical for both leaf and petal growth (Huang & Irish, 2024). In leaves, *TCP5* interacts genetically with *KNAT3* and *SAWTOOTH 1* to control leaf margins and serrations. Their expression domains overlap with the auxin signaling reporter DR5, and RNA‐sequencing data indicate that TCP5 controls auxin signaling, synthesis, and transport (Yu et al., 2021). A similar link between TCP genes and auxin signaling was observed in Arabidopsis petals, where TCP5, 13, and 17 control the switch from cell division to expansion and affect the cuticle ornamentation of the conical cells (van Es et al., 2018). Hence, we propose that TCP control of growth via auxin signaling represents an ancestral mechanism shared by lateral organs. What emerges as a novelty is the discovery that in petals only, TCP5, together with TCP 13 and 17, also inhibits the expression of ethylene biosynthetic genes *ACS2* (*AMYNOCYCLOPROPANE‐1‐CARBOXYLIC ACID SYNTHASE 2*) and *ACC‐OXIDASE 2* while promoting the expression of many *ETHYLENE RESPONSE FACTORS* (*ERFs*) to control the area of the conical cells (van Es et al., 2018). This suggests that the role of ethylene is specific to petal development rather than part of the ancestral toolkit shaping plant lateral organs. It also stresses the significant role that ethylene plays, perhaps more than auxin, in the later stages of petal morphogenesis.

In growing *Arabidopsis* leaves, CK levels are high during the cell proliferation phase and diminish when the transition to the cell expansion stage occurs (Skalák et al., 2019), similar to what was observed in developing rose petals (Wang et al., 2024). TCP4 represses cell proliferation and triggers cellular differentiation in leaves and petals by activating several growth repressor genes, including MYBs and other genes blocking cell‐cycle progression like *ICK1/KRP1*. TCP4 also activates *MIR396b*, which targets *GROWTH REGULATOR FACTOR* (GRFs), positive regulators of leaf growth in maize and Arabidopsis (Lazzara et al., 2024). In leaves, TCP4 interacts with the chromatin remodeling factor BRAHMA to regulate ARR6 and 16, two negative regulators of CK signaling. Because CKs initially maintain the proliferative state and delay leaf cell differentiation, the activation of ARR6 and 16 favors the confinement of the CK‐promoted mitotically active blastozone (the leaf zone where growth takes place) to the marginal portion of the leaf (Efroni et al., 2013). In Arabidopsis petals, *TCP4* expression is regulated by the miR319 and is repressed by the product of the C2H2 zinc finger gene RBE during early petal development to allow petal cell proliferation (Li et al., 2016; Nag et al., 2009; Schommer et al., 2014). So far, no structure comparable to the blastozone has been described in petals, and whether TCP4 acts on petal growth interacting with CK signaling is an important hypothesis for future research. However, “TCP4‐like” factors likely exert a general control of cell proliferation in all plant lateral organs, including stamens (Nag et al., 2009) via the regulation of hormonal pathways. Possibly, the targets of their activity are what differentiate their functions in each organ type.

In maize (*Zea mays*), the mature leaf comprises three morphologically distinct domains: the distal‐most region, the blade, extends away from the stem to capture light; proximally, the sheath wraps around the stem, providing support; the ligule, sandwiched between the sheath and the blade, acts as a protective barrier (Richardson & Hake, 2019). Pre‐patterning of the maize leaf, reminiscent of the pre‐patterns detected in petals, is evident in transcriptomic data from primordium that still appears morphologically uniform, and genes related to hormone metabolism, transport, and signaling are among the differentially expressed genes along the proximo‐distal leaf axis (Leiboff et al., 2021). Laser capture‐RNAseq analyses showed that auxin signaling is higher in the distal part of the leaf primordium during the first positioning of the sheath‐blade boundary. Conversely, genes controlling CK oxidation and negative regulators of CK signaling are upregulated in the pre‐ligule cells, suggesting that low levels of CKs favor cell expansion and the bulging out of the ligule (Leiboff et al., 2021). These observations set a scenario where hormone antagonism defines the proximo‐distal polarity of the leaf primordium and the positioning of developmental boundaries that delimit leaf identity domains. Nonetheless, whether the differential responses and/or distribution of auxin and CK influence axis establishment and boundary pre‐patterning or whether they are set in response to an even earlier, yet unknown, pre‐patterning signal still needs to be addressed.

The apparent uniformity of the maize leaf primordium disappears with the establishment of the pre‐ligule boundary characterized by limited cell expansion, rapid anticlinal cell divisions, and varying cell size (Strable & Nelissen, 2021). Periclinal cell divisions generate a ridge, the first visible morphological mark of the incipient ligule. Then, basally localized cell proliferation and distally localized cell expansion cause a basipetal developmental gradient. The ligule region moves unmodified from the leaf base up to the division and elongation zones. Meanwhile, the leaf undergoes a steady‐state growth phase, transiting from blade to sheath growth and then to the end of growth. A gradient‐like distribution of GA peaks at the pre‐ligule boundary and promotes the mitotic activity; thereby, two CK oxidases and a CK negative regulator belonging to the type‐A ARR class allow the outgrowth of the pre‐ligule (Johnston et al., 2014; Nelissen et al., 2012). How the GA gradient is generated, what triggers CK clearance, and how these hormone dynamics eventually integrate with signals establishing medio‐lateral polarity is unknown (Robil & McSteen, 2023). Equally unclear is whether GAs and CKs exhibit parallel behaviors during the pre‐patterning phase of petal development (see Section 2) and whether those hormones could participate in the specification of the bullseye pre‐pattern boundary in petals (Riglet et al., 2024). In carpels, floral organs also evolved from leaves; axes establishment similarly involves hormone signaling (reviewed in Dong & Østergaard, 2019; Gonçalves, 2021). The medial and lateral gynoecium domains are defined through auxin and cytokinin (CK) crosstalk, which specify axial coordinates. In the incipient gynoecium lateral domain, the auxin response is high and induces the expression of *AHP6* (*ARABIDOPSIS HYS PHOSPHOTRANSFERASE 6*), a CK‐signaling factor that dampens the CKs phosphotransfer signaling cascade and confines it to the medial region of the gynoecium. CKs feedback on auxin by promoting its biosynthesis through YUCCA 1 and YUCCA 4 and by reinforcing polar auxin transport through PIN7 and 3. The mechanisms that establish these domains pattern the emerging gynoecium (Müller et al., 2017). Taken together, these cases suggest that auxin, CKs, and other hormones contribute to the establishment of boundaries and polarity axes in leaves and their derived floral organs, including petals. We anticipate that the ongoing development of ratiometric sensors for an ever‐increasing range of plant hormones (Balcerowicz et al., 2021; Herud‐Sikimić et al., 2021; Rizza et al., 2017; Shi et al., 2024) and their introduction in multiple species should soon allow us to characterize hormone distribution in both leaves and petals with unprecedented spatio‐temporal resolution. This will clarify shared roles and expand the breadth of the foliar theory.

### Hormonal regulation of cell fate specification and differentiation: Parallels between leaves and petals

Hormone signaling also influences cell differentiation events in both organs, likely through mechanisms that are at least in part shared. In Arabidopsis, *BLADE‐ON‐PETIOLE* (*BOP*) genes regulate the growth of the proximal leaf region and the differentiation of the petiole by repressing genes that sustain the meristematic state and cell proliferation (Hepworth et al., 2005). In rice leaves, BOPs promote the differentiation of the proximal sheath and suppress distal blade differentiation, eventually controlling the sheath‐to‐blade ratio (Toriba et al., 2019). Interestingly, two homologues of BOPs were recently found to control corolla growth in wishbone flowers (*Torenia fournieri*) (Su et al., 2023). Mutations in *TfBOP2* result in abnormal petal fusions and defects in proximal corolla differentiation: petals of *tfbop2* mutants are distalized but still produce a tube and a neck indicating *TfBOP2* does not divide petal primordia into different compartments during the pre‐patterning phase but instead promotes the growth and differentiation of the corolla proximal region later in development (Su et al., 2023). The mechanisms restricting TfBOP2 activity to the petal proximal region and the downstream events it triggers are not characterized yet but likely phytohormones are involved.

In *Mimulus verbenaceus*, the R2R3‐MYB gene *STRIPY* is necessary to form a mediolateral anthocyanin stripe in leaves, a trait recently evolved in wild populations (LaFountain et al., 2024) (Figure 3b). Chemical mutagenesis revealed upstream activators and repressors that form a “hidden” prepattern along the leaf proximodistal axis, restricting the expression domain of *STRIPY* (LaFountain et al., 2024). *ELONGATED HYPOCOTYL5* (*HY5*) is expressed everywhere in the leaf and promotes anthocyanin biosynthesis by activating *STRIPY* expression, while *ALOG1* and *TCP5*, expressed in opposing gradients along the proximodistal leaf axis, inhibit *STRIPY* transcription (LaFountain et al., 2024). Hence, leaves, akin to petals, are compartmentalized by pre‐patterning events. These compartments are often concealed but become evident under certain circumstances or following evolutionary events, like the co‐option of *STRIPY* into a pre‐existing GRN, originating the bold pigmentation stripe characteristic of *M. verbenaceus* leaves. We hypothesize that hormone signaling acts upstream of those regulators and constitutes a shared process allowing both leaves and petals to pre‐pattern their surface, while the downstream regulators are organ‐specific and represent derived acquisitions (Figure 3c). Indeed, *M* *imulus* leaves lose their stripe when *STRIPY* is knocked down, but the corolla pigmentation remains unaltered because anthocyanin production is regulated by different *MYB* genes in petals. Similarly, both the spur‐promoting *POP* gene in Columbine and its homologue in Medicago, *PALM1 (PALMATE‐LIKE PENTAFOLIATA1*) regulate compound leaf development (Ballerini et al., 2020; Chen et al., 2010; Ge et al., 2010). Together, these results illustrate how the GRNs that pattern plant lateral organs and facilitate the emergence of petal novelties, like the spur, can involve elements recruited from a pre‐existing leaf GRN and constitute mosaics of old and new (Figure 3c).

## CONCLUSIONS

Despite the conspicuousness of flowering plants corollas, the mechanisms governing their development and evolution still hold many mysteries (Box 1). Petal primordia emerge on the surface of the FM, similarly to simple leaf blades, with elaborations of characteristic petal features and complex shapes occurring later (Fu et al., 2022). To date, most reports support models where hormones act on petal morphogenesis and patterning predominantly through a balancing act impacting growth. On the one hand, the activity of hormone signaling pathways can be restricted to subregions of the petals, allowing phytohormones to govern the shape of each individual petal part and act as pattern modifiers (Galipot et al., 2021). On the other hand, after the early establishment of the developmental boundaries, mechanisms must exist to allow coordinated growth of the different petal domains, and hormones are likely to mediate—at least in part—such synchronization. These models echo findings in the RAM and SAM where auxin, CKs, and GAs regulate cell division, cell differentiation, and the balance between them (Lee et al., 2019; Yamoune et al., 2021). However, to start capturing the juggling act of hormone signaling during petal development, we still need to precise the contribution of each hormonal pathway and connect single hormonal contributions into more extensive networks.

Box 1 **Bullet Point Summary of the Main Points**

- Due to their biochemical properties, versatile and extensive signaling machinery, and ability to be differentially distributed, plant hormones could contribute to all key processes that shape and pattern the corolla of flowering plants: from axes specification to developmental boundaries establishment, compartmentalising the emerging petals into a “paint‐by‐number” canvas.
- Plants hormones are regulators of petal cell fate specification and elaboration, but the molecular pathways supporting these roles remain to be elucidated.
- Phytohormones contribute to petal growth, and the spatio‐temporal dynamics of their activity are key to generate specific shapes and forms.
- Evolutionary tinkering affecting hormonal signaling pathways could contribute to the evolution of a diversity of petal morphologies and patterns.
- Parts of the hormonal networks regulating petal patterning and morphogenesis are shared with those shaping leaves, reflecting their common evolutionary origin.

Overall, the roles hormones play during the earliest phase of petal development, while polarity axes are set up and developmental boundaries are established, appear the least clear, and this represents an exciting area for future research. Exploring how petal polarities are specified across scales and what connections exist between hormone signaling and the following patterning and morphogenetic events during petal development bears the potential to widen our understanding of the link between cell and tissue dynamics, with findings reaching far beyond petal morphogenesis. Hormones participate in epidermal cell differentiation, in the elaboration of specific cell shapes and textures, as well as in the regulation of pigment production. However, most studies to date have focused on the role of auxin during conical cell differentiation in Arabidopsis, and data are lacking (i) to determine whether hormones contribute to the diversification of other petal cell shapes and textures, and (ii) to reach a holistic understanding of hormones contribution to the specification of the physical and chemical features of petal cells.

Equally promising is investigating whether evolutionary modifications of hormonal pathways have contributed to the diversification of corolla forms. Hormone effects on floral morphology could have first evolved as a plastic response to changing environments: as hormones mediate responses to environmental stresses, the emergence of new petal traits as part of a stress response could have been facilitated by adjustments and repurposing of hormone signaling. As some of those new traits likely become adaptive, additional changes in hormonal pathways may have later been selected, enabling the genetic fixation of some of these traits (Wessinger & Hileman, 2020).

We are entering a promising era for floral developmental biology: some of the technical difficulties that—until recently—have limited our ability to investigate petal development and visualize the dynamics of hormone distribution with sufficient spatio‐temporal resolution are being overcome. The advent of new technologies for high‐throughput functional genomics and live imaging linking gene activity to cell behavior holds the promise to revolutionize the field and answer outstanding questions (Box 2). As reviewed here, studies on emerging model systems with diverse anatomies and characteristics have already expanded our understanding of petal morphogenesis, allowing us to identify recurring themes but also ensuring that observations taken in a single species are not too quickly turned into general principles. Increasing the range of models amenable to experimental investigation has also been vital to start (i) investigating the role hormonal signaling plays during the development of floral traits absent from classic models (for example nectar spur), (ii) exploring the contribution of phytohormones to the emergence of novel traits, and (iii) eventually clarifying the part they play in specifying, maintaining, or modifying the dimensions of petal colorful patterns. It is now essential that new technical advancements and protocols developed for Arabidopsis are adapted to and deployed into this new set of emerging model systems. The Pandora's box of petal evo‐devo is now open, and a flurry of exciting discoveries is just waiting to be released.

Box 2 **Open Questions and Perspectives**

- *Question 1: Hormones as boundary builders?* Can plant hormones establish developmental boundaries across petal primordia and, if so, what are the molecular mechanisms at play?
- *Question 2: Hormones as coordinators or modifiers?* How can plant hormones coordinate growth between different petal regions to conserve pattern proportions as petals develop or regulate the growth of different petal subdomains independently, acting instead as pattern modifiers?
- *Question 3: Hormones as integrators?* To which extent are petal patterning and morphogenesis influenced by interactions among hormone signaling pathways and other types of small molecules like miRNA?
- *Question 4: Hormones as team players?* What are the relative contributions of different hormones to petal morphogenesis and patterning, and how much does hormonal crosstalk matter?
- *Question 5: Hormones as recycled agents or forces for changes?* How much of the hormonal pathways that shape petals reflects inheritance from ancestral networks at work in other plant lateral organs, and have changes affecting phytohormone signaling facilitated the emergence of unique morphological novelties in the corolla of angiosperms?

## Data Availability

Data sharing is not applicable to this article as no new data were created or analyzed in this study.
